# Patterns of health service use in community living older adults with dementia and comorbid conditions: a population-based retrospective cohort study in Ontario, Canada

**DOI:** 10.1186/s12877-016-0351-x

**Published:** 2016-10-26

**Authors:** Lauren E. Griffith, Andrea Gruneir, Kathryn Fisher, Dilzayn Panjwani, Sima Gandhi, Li Sheng, Amiram Gafni, Christopher Patterson, Maureen Markle-Reid, Jenny Ploeg

**Affiliations:** 1 Department of Clinical Epidemiology and Biostatistics, McMaster University, McMaster Innovation Park, 175 Longwood Road South, Hamilton, ON L8P 0A1 Canada; 2 Department of Family Medicine, 6-40 University of Alberta, 6-10 University Terrace, Edmonton, AB T6G 2T4 Canada; 3 School of Nursing, McMaster University, Health Sciences Centre, 1280 Main Street West, Room 3N25B, Hamilton, ON L8S 4K1 Canada; 4 Women’s College Research Institute, Women’s College Hospital, 790 Bay St., 7th floor, Toronto, ON M5G 1N8 Canada; 5 Institute for Clinical Evaluative Sciences, 2075 Bayview Avenue, Toronto, ON M4N 3M5 Canada; 6 Centre for Health Economics and Policy Analysis; Department of Clinical Epidemiology and Biostatistics, McMaster University, 1280 Main Street West, Room CRL-208, Hamilton, ON L8S 4K1 Canada; 7 Department of Medicine, McMaster University, Health Sciences Centre, 1280 Main Street West, Room 3N25B, Hamilton, ON L8S 4K1 Canada

**Keywords:** Dementia, Community-living older adults, Health service utilization, Health service costs, Comorbidity

## Abstract

**Background:**

Patients with dementia have increased healthcare utilization and often have comorbid chronic conditions. It is not clear if the increase in utilization is driven by dementia, the comorbidities or both. The objective of this study was to describe the number and types of comorbid conditions in a population-based cohort of older adults with dementia and how the level of comorbidity impacts dementia-related and non-dementia-related health service utilization.

**Methods:**

This study is a retrospective cohort study using multiple linked administrative databases to examine health service utilization and costs of 100,630 community-living older adults living with pre-existing dementia in Ontario, Canada. Comorbid conditions and health service utilization were measured using administrative data (physician visits, emergency department visits, hospitalizations, and homecare contacts).

**Results:**

Nearly all, 96.3 %, had at least one comorbid condition, while 18.4 % had five or more comorbid conditions. The most common comorbid conditions were hypertension (77.8 %), and arthritis (66.2 %). All types of utilization increased consistently with the number of comorbid conditions. The average number of dementia-related services tended to be similar across all levels of comorbidity while the average number of non-dementia related visits tended to increase with the level of comorbidity.

**Conclusions:**

Comorbidities in community-living older adults with dementia are common and account for a substantial proportion of health service use and costs in this population. Our results suggest that comprehensive programs that take a holistic view to identify the needs of patients in the context of other comorbidities are required for persons with dementia living in the community.

**Electronic supplementary material:**

The online version of this article (doi:10.1186/s12877-016-0351-x) contains supplementary material, which is available to authorized users.

## Background

Worldwide there are an estimated 47.5 million people with dementia, and in 2010, the total healthcare cost for this population was more than 1 % of the global gross domestic product [1, 2]. In 2011, almost 750,000 Canadians were living with dementia, with Alzheimer’s disease being the most common form. [3] Alzheimer’s disease is also among the top ten diseases contributing to deaths and years of life lost in the United States [4]. People diagnosed with dementia tend to have higher health service use, including physician visits and hospitalizations, than those not diagnosed with dementia [5–7]. About half of people with dementia live in the community [8], and the number is increasing [9]. In this group there is a great reliance on informal care, in which family and friends are called upon to provide the majority of care [10]. This is often supported by home-care and community-based care services to provide effective continuing care for those people living in the community with dementia. With the demographic shift toward older populations, the number of people with dementia is projected to increase dramatically over the next 25 years which will impact both formal and informal care needs [11].

Older adults are also at increased risk for many other chronic conditions. Multi-morbidity has been increasingly recognized as an independent risk factor for decreased quality of life [12], increased disability [13] and premature mortality [14]. In people with dementia, comorbid conditions can accelerate cognitive [15] and functional [16] decline. As well, medications or treatments for other conditions may affect cognitive decline [17] especially when polypharmacy is associated with multimorbidity [18]. Thus one may expect dementia-related health service use to increase with comorbidity. Dementia may also complicate clinical care for other chronic conditions and negatively influence the quality of care received [19]. For example, dementia may impair one’s awareness of symptoms, capacity for self-management, and ability to engage in health maintenance activities for other chronic conditions [20]. Thus in patients with dementia and comorbidity, one could speculate that non-dementia-related health service use may also increase. Although health service use is not a sufficient proxy for need [21], it does reflect burden to the healthcare system. From a health services and policy perspective, research suggests that multi-morbidity be considered in the allocation of healthcare resources because estimates of future use of healthcare services are not well summarized by simply totaling the use of services by individual illnesses [22].

Although there are a number of studies describing comorbidity [15, 19, 23, 24] and increased health service utilization and costs [7, 25–28] in people with dementia, there are only a few population-based studies characterizing both comorbidity and health service utilization in community-living people with dementia, and none to our knowledge explore if the increase in utilization is driven by dementia, the comorbidities or both. The objectives of this study are to: 1) describe the number and types of comorbid conditions in a population-based cohort of older adults with dementia; 2) examine how the level of comorbidity impacts both dementia-related and non-dementia-related health service utilization and costs over a 5-year period.

## Methods

### Study design

This is a retrospective cohort study using multiple linked administrative databases to examine health service utilization and costs of a population-based cohort of community-living older adults with dementia.

### Setting

This study is set in Canada’s most populous province, Ontario, with approximately 13 million residents. The vast majority of Ontarians are covered under the provincial health insurance plan (OHIP). Coverage includes outpatient physician visits, acute care hospital use (both in the emergency department and inpatient admissions), homecare, and outpatient prescription drug coverage for those 65 years and older. In Ontario, Community Care Access Centres provide publicly funded homecare, using a contractual model of service delivery, wherein publicly funded case managers contract out homecare services to community agencies that provide care to clients.

### Data

The administrative databases linked in this study include: the Registered Persons Database (RPDB) for basic demographic data on all individuals enrolled in the provincial insurance program; the OHIP claims database for physician visits; the Discharge Abstract Database (DAD) for all records of inpatient hospitalizations; the National Ambulatory Care Reporting System (NACRS) for all records of emergency department visits and other ambulatory contacts; the Same Day Surgery (SDS) database for same-day surgeries and procedures, the Home Care Database (HCD) for information on all homecare service records; and the Ontario Drug Benefits (ODB) claims database for all outpatient prescription claims. Additional data sources were accessed to obtain specific diagnostic information. These included the Ontario Mental Health Reporting System (OMHRS), the Ontario Cancer Registry (OCR), and the Ontario Diabetes Database (ODD). The data were linked using unique, encoded identifiers and analyzed at the Institute for Clinical Evaluative Sciences (ICES) in Toronto, Ontario. These data are regularly used for research purposes and have been studied extensively for their validity [29–34].

### Study cohort

The cohort consists of all individuals aged 66 and over, who resided in the community, and had an existing diagnosis of dementia as of April 1, 2008 (baseline). We set our lower age limit at age 66 to have at least 1 year of available prescription claims, which was necessary to identify some chronic conditions. We defined dementia as the presence of at least 1 diagnostic code in the OHIP claims within the 5 years prior to baseline, or one International Classification of Disease (either version 9 or 10 depending on year) in DAD or NACRS within the 5 years prior to baseline, or 1 claim in the ODB for a cholinesterase inhibitor in the year prior to baseline. This definition of dementia has been used in other population-based studies that have used administrative databases [35] (Additional file 1). We required that any relevant claims or codes be identified prior to October 2007 to ensure that individuals had the condition for at least 6 months at baseline, which was part of our definition of a chronic condition [36]. Individuals who were 105 years or older, receiving palliative care (in any setting), residing outside of Ontario, or had no contact with the health system in the 5 years prior to the baseline date were excluded. We also excluded individuals residing in long-term care homes since they tend to have different patterns of health service utilization than community-living older adults. From baseline, each individual was followed until the first of: admission into a long-term care home, death, a move out of province, or the end of the 5-year follow-up period.

### Comorbid conditions

The comorbid chronic conditions identified were: anxiety/depression, arthritis, cancer, chronic obstructive pulmonary disease (COPD), diabetes, upper gastrointestinal bleed, hypertension, ischemic heart disease, liver disease, osteoporosis/osteopenia, inflammatory bowel disease, renal disease (with and without chronic dialysis), stroke, and other cerebrovascular disease. The list was chosen to utilize pre-existing validated ICES disease algorithms and registries. Each condition was defined using either of the following methods: 1) a search for diagnostic codes in any of the OHIP, DAD, or NACRS databases and/or specification prescription claims within the 5 years prior to baseline; or 2) entry into a diagnosis-specific database created at ICES (Additional file 1). Comorbidity status was characterized by the number of comorbid conditions at baseline (0, 1, 2, or 3 or more).

### Health services utilization

We identified all publically-funded health services utilization during the study period. This included physician visits (both primary care and specialist), unplanned emergency department visits, hospitalizations, and homecare contacts. We identified hospitalization “episodes” so that transfers between hospitals were not counted as separate events. For each hospitalization episode, we estimated the total length of stay (number of days from admission to final discharge), the total number of days in the intensive care unit (ICU), and total number of days designated as Alternate Level of Care (ALC). ALC refers to periods of time when a patient is considered to no longer require hospital-level care but cannot be discharged due to a lack of alternative services (for example, when a long-term care bed is required but not available). This is particularly relevant in dementia patients since it is the most frequent diagnosis associated with ALC designation [37]. We distinguished physician visits, emergency department visits, and hospitalizations for dementia (index condition) from those for all other reasons (non-index conditions). For homecare services, we counted the total number of visits and specific visit types (including case management, nursing, in-home support, and therapies) for cost analyses, but focused on nursing visits for utilization as they are the most common type of service. We were unable to distinguish between homecare services for index and non-index reasons.

### Statistical methods

We described the cohort at baseline by age, sex, neighbourhood income quintile, and the number and type of comorbid chronic conditions for the entire cohort, for those with complete 5-year follow-up, and for those with less than 5 years of follow-up (most often due to death or institutionalization). For each year of follow-up, we estimated the total amount of each type of health service use (physician services, acute care, and homecare) by baseline comorbidity status. Costs for each type of health service were calculated by multiplying the volume of service (either total number of visits or hours of service, depending on type) by the published unit cost (cost per visit or hour, depending on type) (Additional file 2). Total service costs for each year in the 5-year follow-up were calculated by adding the total costs per type. Average annual per patient costs were estimated by dividing total service costs by the number of individuals in the cohort at the beginning of the year. We also estimated the proportion of total health service costs attributed to physician visits, emergency department visits, hospitalizations, and homecare. All costs were expressed in 2012 Canadian (CDN) dollars. Because the main intent of this study was to present descriptive statistics on the full population of community-living older adults with dementia in Ontario, the decision was made not to use statistical tests given the large cohort size which would have resulted in small p-values.

## Results

### Comorbidity in older adults with dementia

We identified 100,630 community-living older adults with pre-existing dementia, representing 6 % of the Ontario population aged 66 years and older, as of April 1, 2008 (Table 1). Approximately 20 % were aged 66–74 years, 48 % were 75–84 years, and 32 % aged 85 or over. Women comprised about 60 % of the cohort. The average number of comorbid conditions was 2.9, which did not vary across age groups. Nearly all, 96.3 %, had at least 1 comorbid condition, while 18.4 % had 5 or more comorbid conditions. The most common comorbid conditions were hypertension (77.8 %), arthritis (66.2 %), COPD (28.4 %) and diabetes (28.0 %). Over half of the cohort could not be followed for the full 5 years, mainly due to death (35.8 %) or institutionalization (16.0 %) during that time. People followed for the full 5 years tended to be younger and have fewer chronic conditions (Table 1).

**Table 1:** Baseline characteristics of community-dwelling older adults in Ontario living with dementia

	Dementia cohort (*n* = 100,630)	Amount of follow-up	
		5 years (*n* = 48,458)	<5 years (*n* = 52,172)
Age groups (n, %)			
66–69	6958 (6.9 %)	5186 (10.7 %)	1772 (3.4 %)
70–74	13,421 (13.3 %)	8861 (18.3 %)	4560 (8.7 %)
75–79	21,260 (21.1 %)	11,755 (24.3 %)	9505 (18.2 %)
80–84	26,677 (26.5 %)	12,252 (25.3 %)	14,425 (27.6 %)
85–89	20,833 (20.7 %)	7374 (15.2 %)	13,459 (25.8 %)
90+	11,481 (11.4 %)	3030 (6.3 %)	8451 (16.2 %)
Female (n, %)	60,964 (60.6 %)	29,357 (60.6 %)	31,607 (60.6 %)
Income quintile (n, %)			
1 (Lowest)	21,689 (21.6 %)	10,027 (20.7 %)	11,662 (22.4 %)
2	20,654 (20.5 %)	9987 (20.6 %)	10,667 (20.4 %)
3	19,273 (19.2 %)	9148 (18.9 %)	10,125 (19.4 %)
4	18,739 (18.6 %)	9152 (18.9 %)	9587 (18.4 %)
5 (Highest)	19,763 (19.6 %)	9958 (20.5 %)	9805 (18.8 %)
Number of comorbid conditions (n, %)			
0	3750 (3.7 %)	1997 (4.1 %)	1753 (3.4 %)
1	13,415 (13.3 %)	7116 (14.7 %)	6299 (12.1 %)
2	23,543 (23.4 %)	12,398 (25.6 %)	11,145 (21.4 %)
3	23,923 (23.8 %)	11,924 (24.6 %)	11,999 (23 %)
4	17,462 (17.4 %)	8032 (16.6 %)	9430 (18.1 %)
5	18,537 (18.4 %)	6991 (14.4 %)	11,546 (22.1 %)
Comorbid conditions (n, %)			
Hypertension	78,246 (77.8 %)	36,962 (76.3 %)	41,284 (79.1 %)
Arthritis	66,601 (66.2 %)	32,427 (66.9 %)	34,174 (65.5 %)
Chronic Obstructive Pulmonary Disease	28,557 (28.4 %)	12,277 (25.3 %)	16,280 (31.2 %)
Diabetes	28,187 (28 %)	13,289 (27.4 %)	14,898 (28.6 %)
Ischemic Heart Disease	24,075 (23.9 %)	10,527 (21.7 %)	13,548 (26.0 %)
Inflammatory Bowel Disease	22,532 (22.4 %)	9946 (20.5 %)	12,586 (24.1 %)
Cancer	17,981 (17.9 %)	7616 (15.7 %)	10,365 (19.9 %)
Osteoporosis/osteopenia	10,162 (10.1 %)	3449 (7.1 %)	6713 (12.9 %)
Renal Disease	10,153 (10.1 %)	3799 (7.8 %)	6354 (12.2 %)
Anxiety and/or Depression	6411 (6.4 %)	2723 (5.6 %)	3688 (7.1 %)
Stroke	6294 (6.3 %)	2524 (5.2 %)	3770 (7.2 %)
Upper Gastrointestinal Bleed	3862 (3.8 %)	1454 (3.0 %)	2408 (4.6 %)
Cerebrovascular Disease	3732 (3.7 %)	1476 (3.0 %)	2256 (4.3 %)
Liver Disease	774 (0.8 %)	301 (0.6 %)	473 (0.9 %)

### Health service utilization and costs

**Fig. 1:**
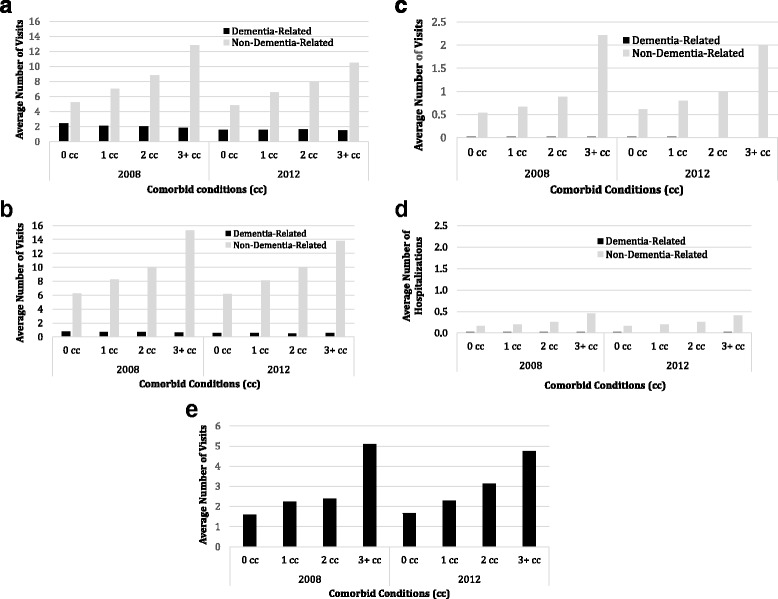
**a** Average annual number of dementia-related and non-dementia-related General practitioner visits. **b** Average annual number of dementia-related and non-dementia-related specialist visits. **c** Average annual number of dementia-related and non-dementia-related emergency department visits. **d** Average annual number of dementia-related and non-dementia-related hospitalizations. **e** Average annual number of dementia-related and non-dementia-related homecare nursing visits by the number of comorbid conditions in 2008 and 2012. Black bars represent dementia-related utilization and grey bars represent non-dementia-related utilization

Figure 1a-e displays the average annual number of physician visits (a:primary care and b:specialist), c:emergency department visits, d:hospitalizations, and e:homecare visits by the number of comorbid conditions for 2008 and 2012. For all services except homecare visits, dementia-related and non-dementia-related use is shown. All types of utilization increased with the number of comorbid conditions. In most cases the 3+ comorbid conditions group had at least twice the utilization compared to those with no comorbidities. At all levels of comorbidity the vast majority of services were attributed to non-dementia reasons. The average number of dementia-related services tended to be similar across all levels of comorbidity while the average number of non-dementia related visits tended to increase with the level of comorbidity. For all types of services, there was little change over the 5 years of follow-up, except for a slight decrease in the average annual number of services in those with three or more comorbidities.

**Fig. 2:**
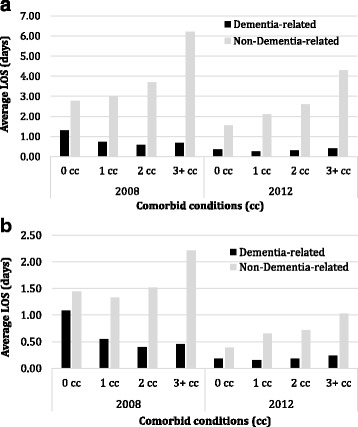
**a** Average length of stay (LOS) for dementia-related and non-dementia-related medical or surgical hospital episodes by the number of comorbid conditions in 2008 and 2012. **b** Average length of stay (LOS) for dementia-related and non-dementia-related alternate level of care (ALC) hospital episodes by the number of comorbid conditions in 2008 and 2012. Black bars represent dementia-related LOS and grey bars represent non-dementia-related LOS

There was a similar trend of increasing hospital length of stay (LOS) for medical and surgical episodes (Fig. 2a) and for ALC episodes (Fig. 2b) with number of comorbidities for non-dementia-related use. Dementia-related LOS tended to be shorter with increasing level of comorbidity. In 2008, the average LOS for acute care episodes for non-dementia reasons increased with the number of comorbidities (2.77 days in those with 0 comorbidities to 6.21 days in those with 3+ comorbidities). The average LOS for acute care episodes associated with dementia was the highest for those with 0 comorbidities (1.33) and lower for those with one or more comorbidities (0.74 for 1, 0.59 for 2 and 0.67 for 3+). Overall the average LOS decreased over time for both dementia and non-dementia related episodes and for all levels of comorbidity.

**Fig. 3:**
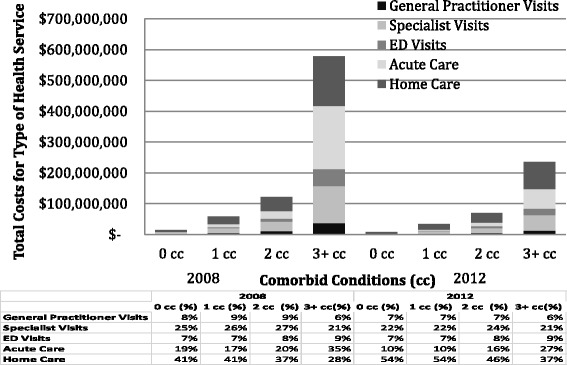
Total health services costs in 2008 and 2012 (expressed in 2012 CDN dollars) by level of comorbidity and by service type

Figure 3 displays the total costs associated with health service use in 2008 and 2012 (expressed in 2012 CDN dollars) by level of comorbidity and by service type. Total health services costs in 2008 increased sharply with the number of comorbid conditions from $13,000,000 in those with 0 to $532,000,000 in those with 3 or more comorbid conditions. Among those with 0 comorbid conditions, the main cost driver was homecare but this was replaced by acute care episodes among those with three or more conditions. Overall cohort costs declined by 2012 (due to attrition) but the general trends in terms of the relative drivers of costs at different levels of comorbidity persisted.

**Fig. 4:**
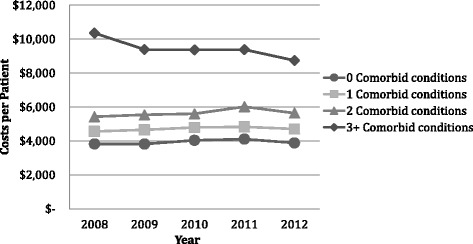
Per patient health service use costs over 5 years (2008 to 2012) adjusted to 2012 CDN dollars for patients with 0, 1, 2, or 3 or more comorbid conditions (CC)

The average annual per patient costs increased with the number of comorbid conditions from $3467 in those with 0, to $8873 in those with three or more in 2008 (Fig. 4). Overall average per patients costs, adjusted to 2012 CDN dollars, declined due to decreased utilization over the 5 years but the decline was not consistent at each level of comorbidity. For those with 0 comorbid conditions the costs stayed the same, for 1–2 comorbid conditions the average per patient costs increased by about $200 over time, whereas those with 3+ declined by $1600.

## Discussion

This study makes an important contribution to our understanding of health-system burden of community living older adults with dementia. Like many studies, we found that health service use increased substantially with the number of comorbidities, with those with three or more comorbid conditions seeing a GP and specialist more than once a month on average. In a systematic review by Lehnert et al. [38] the authors reported that almost all studies observed a positive association of multiple chronic conditions and health service use and costs, and that several studies found a near exponential relationship between the number of chronic conditions and costs.

We also wanted to examine how the level of comorbidity impacts both dementia-related and non-dementia-related health service utilization, as one could make a case that either one or both could increase as comorbidity increases. We found that overall health service use and costs increased with number of comorbidities, but this was driven mostly by non-dementia-related use. In fact, even in people with no other comorbidities, we found that non-dementia-related use was greater than dementia-related use. The dramatic increase in non-dementia related services may indicate that dementia is negatively impacting self-care and management for other chronic conditions. This could be exacerbated by the under-diagnosis of chronic conditions in older adults [39] especially in those with dementia [40]. However, it may also be partially explained by our data source. For example, for hospitalizations we used the “most responsible diagnosis”, which is based on the diagnosis considered to have most contributed to the overall length of stay, to determine if a hospital episode was dementia-related or non-dementia-related. This could happen if dementia is accelerating other chronic conditions (i.e., impacting non-dementia-related health service use) or increases the likelihood of other acute events, such as falls or pneumonia, the hospitalization may not be directly attributed to dementia or captured in the comorbid conditions that we counted. The most common reasons for hospitalizations for people with AD are syncope, falls and trauma, which would not likely be counted as “dementia-related use” in these analyses [3].

Hypertension and arthritis were the most common comorbidities in the dementia cohort. In a recent systematic review of disease clusters in older adults, Sinnige et al [41] found that hypertension and stroke were the most common diseases to cluster with dementia, although they reported that dementia was considered in only one-fifth of the studies included in the review. Our results are similar to the most common combinations of chronic conditions found in the general population [42]. In a systematic review of patterns of comorbidity in primary care, Violen et al. [43] reported that osteoarthritis and cardiovascular and/or metabolic conditions was the most common combination of chronic conditions. When comparing dementia patients and non-dementia patients in primary care, Schubert et al. [24] found that comorbidity profiles were similar in the two groups, however Bynum et al. [26] did not.

We found that the drivers of costs differed in people with no comorbidity compared to those with three or more chronic conditions. In those with no comorbidity, the biggest driver was homecare and in those with three or more chronic conditions, the biggest driver was acute care episodes. In a 3-year follow-up study, Rudolph et al. [44] found that 66 % of persons with Alzheimer’s disease were hospitalized at least once and 47 % were hospitalized two or more times. Furthermore, even after adjusting for age, gender and other potential confounders, Phelan et al. [6] found that dementia patients were more likely to be hospitalized than non-dementia patients (rate ratio 1.41; 95 % CI 1.23–1.61) especially for ambulatory care-sensitive conditions for which proactive outpatient care may prevent the need for hospital stay (rate ratio 1.78; 95 % CI 1.38–2.31). Similar results were reported by Feng et al. [45] who found that community-living people with dementia were more likely to have potentially preventable hospitalization, an emergency room visit that was potentially avoidable, and an emergency department visit that resulted in a hospitalization, than those without dementia. Our results suggest that this phenomenon occurs increasingly with the level of comorbidity.

The impact of comorbidity on utilization is a particularly important issue for ALC days. The estimates of the percent of ALC patients with dementia range between 25 % [46] to over 60 % [37]. The use of hospital beds for ALC patients can contribute to a decrease in acute care capacity, emergency department overcrowding, and patient flow inefficiencies throughout the entire healthcare system [47]. We found that overall in older adults with dementia that the ALC LOS increased with level of comorbidity, but this again was mostly driven by non-dementia-related hospital stays. This suggests that the impact of dementia on ALC days may be under-estimated in administrative-data based studies and underscores the complexity of these patients. Most ALC patients are waiting for placement in long-term care facilities [37, 48]. A study exploring ALC patients waiting for nursing home admission, however, found that some of these patients could be discharged to a community setting with the support of transitional programs and increased community care [49]. As most people with dementia want to stay at home for as long as possible [50], this would require a greater integration of care between the acute and post-acute care providers [51].

Using comprehensive administrative data, this study represents all community-living older adults in Ontario with dementia. However, this study has limitations. We follow a cohort over time, but have restricted the assessment of comorbidity to the baseline. As we would not be capturing new comorbidities over the 5 years, this would tend to underestimate the impact of comorbidity on utilization. For example, those with 0 comorbidities at baseline could have three or more comorbidities by year 5. As well, we included a restricted list of comorbidities to 14 conditions. This list does, however, include the most common combinations of conditions identified in Canada [42] as well the most common conditions included in multi-morbidity research [52]. Although we were able to present a snapshot of community living older adults with dementia in our cohort, we lacked information on their degree of cognitive impairment. The degree of cognitive impairment is related to healthcare utilization and costs [25] and impacts the ability to self-manage other chronic conditions. We excluded medications from our estimates of utilization and costs. Although medications are an important aspect of healthcare expenditures in older adults, research suggests they contribute a relatively lower proportion of overall healthcare costs in people with dementia. In a study of Medicare beneficiaries, those with Alzheimer’s disease or dementia had higher average annual per-person payments for all healthcare services (hospital, physician and other medical providers, nursing home, and home healthcare) except for prescription medications [3]. Because of our health-service perspective, we did not include utilization or costs associated with informal care which are significant in older adults with dementia [3, 53]. Finally, our classification of health service use into dementia-related and non-dementia-related is based on the most responsible condition. In cases where utilization was for the treatment of sequelae of dementia (e.g., falls or delirium), it may not be counted as dementia-related and thus underestimate dementia-related use. We did, however, find a consistent trend over all types of health service use and different administrative data sources.

## Conclusions

These data reflect the totality of publicly-funded health services provided for a cohort of community-living older adults with dementia in Ontario, the most populous province of Canada. We found that the majority of community-living older adults with dementia have multiple co-existing chronic conditions which are strongly associated with increased healthcare service utilization and costs. While increased utilization and associated costs may not reflect actual need or optimal use [21], they nevertheless reflect burden and suggest areas where opportunities exist to reduce burden. For example, homecare services targeting prevention and health promotion could reduce the use of expensive acute care services, particularly those that are unplanned and/or potentially avoidable [45]. In addition, older adults with dementia frequently experience care transitions [5, 24], which require an integration of services to provide effective continuing care. Our results suggest that policy makers should direct resources to comprehensive programs that take a holistic view to identify the needs of patients in the context of other comorbidities and are tailored to the complexity of care required for persons with dementia living in the community [54].

## Additional files

Additional file 1: Appendix 1. Diagnostic Definitions for Index Condition (Dementia) and Co-morbid Conditions. (PDF 201 kb)
Additional file 2: Appendix 2. Costing Methods and Data Sources. (PDF 83 kb)

## Abbreviations

ALC: Alternate Level of Care
CC: Comorbid condition
CIHI: Canadian Institute for Health Information
CIHR: Canadian Institutes of Health Research
COPD: Chronic obstructive pulmonary disease
DAD: Discharge Abstract Database
HCD: Home Care Database
ICES: Institute for Clinical Evaluative Sciences
ICU: Intensive care unit
LOS: Length of stay
NACRS: National Ambulatory Care Reporting System
OCR: Ontario Cancer Registry
ODB: Ontario Drug Benefits
ODD: Ontario Diabetes Database
OHIP: Ontario Health Insurance Plan
OMHRS: Ontario Mental Health Reporting System
RPDB: Registered Persons Database
SDS: Same Day Surgery
